# Heart rate variability is associated with interstitial glucose fluctuations in type 2 diabetic women treated with insulin

**DOI:** 10.1186/s40064-016-1932-z

**Published:** 2016-03-15

**Authors:** Vadim V. Klimontov, Natalia E. Myakina, Nadezda V. Tyan

**Affiliations:** Laboratory of Endocrinology, Scientific Institute of Clinical and Experimental Lymphology, Timakov Str., 2, Novosibirsk, Russian Federation 630060

**Keywords:** Type 2 diabetes, Heart rate variability, Hypoglycemia, Glucose variability, Cardiovascular autonomic neuropathy

## Abstract

Heart rate variability (HRV) analysis is a commonly used tool for assessment of autonomic function in diabetic subjects. Nevertheless, the effects of glucose fluctuations on HRV remain to be clarified. In this study we investigated the associations of frequency-domain HRV parameters with current and antecedent interstitial glucose fluctuations in insulin-treated type 2 diabetic women at high cardiovascular risk. Sixty-seven women with type 2 diabetes, from 48 to 78 years of age, including 46 ones with cardiovascular autonomic neuropathy (CAN), underwent simultaneous continuous glucose monitoring (CGM) and Holter recording. Eight glucose variability (GV) indices, including standard deviation, 2-h continuous overlapping net glycemic action (CONGA2), lability index, J-index, mean amplitude of glucose excursions, mean absolute glucose (MAG), low blood glucose index (LBGI) and high blood glucose index (HBGI), were calculated from CGM data. The low frequency (LF) and high frequency (HF) power values were estimated on 5-min intervals at fasting and postprandial daytime periods, at night and during CGM-defined hypoglycemia. The values of LF and HF power declined after meals in diabetic women with normal autonomic function tests. Patients with CAN demonstrated blunted postprandial LF and HF reduction and diminished LF/HF ratio during daytime hypoglycemic events. Daytime LF and HF at fasting state correlated negatively with MAG derived from antecedent nocturnal CGM recordings. Positive correlation was found between fasting LF and nocturnal LBGI. The LF power during daytime hypoglycemia demonstrated negative correlations with nocturnal CONGA2, J-index, HBGI and MAG. The nocturnal HBGI and CONGA2, along with HbA1c and daily insulin dose, were predictors of LF during daytime hypoglycemia in multiple regression analysis. Both postprandial and antecedent nocturnal glucose fluctuations affect daytime frequency-domain HRV parameters in insulin-treated type 2 diabetic women. In patients with increased GV the results of short-term assessment of HRV should be interpreted with caution. Fasting state rather than postprandial one seems to be preferable for HRV estimation.

## Background

Heart rate variability (HRV) analysis is a commonly used tool for assessment of autonomic function. The presence of reduced HRV in patients with diabetes has been attributed to cardiac autonomic impairment, which appears early in hyperglycemia settings (Tarvainen et al. 2014; Ziegler et al. 2015). Importantly, the reduced HRV is associated with increased mortality in type 2 diabetic subjects at high cardiovascular risk (Pop-Busui et al. 2010).

The emerging evidence suggests that glycemic variability (GV) may be associated with autonomic imbalance and reduced HRV in both type 1 and type 2 diabetes (Fleischer 2012; Jaiswal et al. 2014). Previously it was demonstrated that acute glycemic excursions can modify the results of autonomic cardiovascular tests in both healthy and diabetic individuals (Tarvainen et al. 2014; Schächinger et al. 2004; Koivikko et al. 2005; Vlcek et al. 2008; Nguyen et al. 2013; Soydan et al. 2013; Limberg et al. 2014). The situation may be even more confusing due to prolonged effect of antecedent hypoglycemia on autonomic function (Segel et al. 2002; Adler et al. 2009; Moheet et al. 2014). The impact of current and antecedent glucose excursions on HRV requires further investigations.

Simultaneous continuous glucose monitoring (CGM) and Holter recording provides the excellent opportunity for the study of relationships between glucose fluctuations and HRV. However, current evidences of such relationships in type 2 diabetes are limited (Di Flaviani et al. 2011; Kalopita et al. 2014; Fleischer et al. 2015). The relationships between HRV and GV indexes in insulin-treated type 2 diabetic patients have not been studied yet.

In this work we investigated the associations of frequency-domain HRV parameters with current and antecedent interstitial glucose fluctuations in insulin-treated type 2 diabetic patients at high cardiovascular risk.

## Methods

### Subjects

Sixty-seven Caucasian type 2 diabetic women, from 48 to 78 years of age (median 65 years), were recruited. Taking into account the sex differences in cardiac autonomic modulation in patients with type 2 diabetes (Fleischer et al. 2015), we enrolled women only in this study. Type 2 diabetes, diagnosed according to WHO (2006), documented diabetes duration ≥5 years, and current insulin treatment were used as other main inclusion criteria.

The exclusion criteria were set in order to minimize the burden of confounding factors, affecting the HRV (Spallone et al. 2011a, b). Thereafter, subjects with congestive heart failure, pulmonary diseases, estimated glomerular filtration rate (eGFR [CKD-EPI 2009]) <30 mL/min/1.73 m^2^, those on psychoactive drugs or sympatholytics other than β-blockers, were not included. Due to limitations in HRV analysis, subjects with atrial fibrillation, ventricular extrasystoles (2–5 Lown–Wolf classes), atrioventricular block or other clinical significant arrhythmias were not included also.

Most of the subjects received basal-bolus insulin therapy (n = 36), other ones were treated by premixed insulins (n = 13) or basal insulin only (n = 18). In most cases insulin was combined with oral antihyperglycemic agents, including metformin (n = 39), sulfonylurea (n = 9) or dipeptidyl peptidase-4 inhibitors (n = 4). The values of hemoglobin A1c (HbA1c) exceeded the individual target levels in 45 patients.

To standardize the conditions, all participants were admitted to the hospital for a short time for research purposes. During CGM and ECG recordings, all participants received a standardized diet (about 75 g of protein, 60 g of fat and 280 g of carbohydrates daily), with fixed meal-time. Patients had their breakfast, lunch and dinner at 08:30, 13:30 and 17:30, respectively. No medical procedures, except injections of regular agents, were performed during the study.

### Glucose monitoring and GV assessment

Blinded CGM lasting for at least 48 h was performed using *Medtronic MiniMed iPro2* system (*iPro™2 digital recorder, MMT*-*7741*) with an *Enlite™* sensor *(MMT*-*7008)*. Sensors were inserted in all patients using the *Enlite Serter(MMT*-*7510).* At least five (normally six) blood glucose readings per day were obtained from each patient by *One Touch*^*®*^*Verio Pro* + Blood Glucose Meters (*LifeScan, Inc.*). Patients were asked to record their meals, blood glucose, activities and medications during CGM.

All CGM data were uploaded into the online system (*CareLink iPro™ Therapy Management Software for Diabetes, MMT*-*7340*), through the *iPro™2 Docking Station(MMT*-*7742 or Dock).* After the uploading, the meter readings and any other recorded events were manually entered into *CareLink iPro* to calibrate the sensor data. All the patients’ reports were viewed individually to find and eliminate calibration errors. Then all the CGM data were exported from *CareLink iPro online system* as a character-separated values (CSV) file. These CSV data were manually processed for further GV calculating.

The mean CGM duration was 74.9 (54.4; 93.7) h. The data from initial 2 h of monitoring, which is considered to be an unstable calibration period, were excluded from analysis (Hirsch et al. 2008).

Hypoglycemia was defined as an episode of interstitial glucose ≤3.9 mmol/L (≤70 mg/dL). The episodes with a minimum duration of 20 min were considered to be clinically significant (UK Hypoglycaemia Study Group 2007).

Based on CGM data, mean glucose and 8 intraday GV indices, including standard deviation (SD), 2-h continuous overlapping net glycemic action (CONGA2), lability index, J-index, mean amplitude of glucose excursions (MAGE), mean absolute glucose (MAG), low blood glucose index (LBGI) and high blood glucose index (HBGI), were estimated. The clinical significance of these indices had been reviewed recently (Service 2013; Klimontov and Myakina 2014; Suh and Kim 2015). In brief, SD, LI, MAGE and MAG reflect the GV in general, HBGI, J-index and CONGA2 associated with hyperglycemic fluctuations predominantly, while LBGI is most sensitive to hypoglycemia. The indices were computed with EasyGV calculator (version 9.0) (Hill et al. 2011). Most of GV indices were calculated for nocturnal hours (0:00–5:59) and daytime hours (6:00–23:59) separately. MAGE was calculated for daytime only.

### Holter monitoring and autonomic function evaluation

Patients underwent continuous ECG monitoring for 24 h. The ECG recordings were initiated between 08:30 and 09:30 a.m., after 12–16 h from the start of CGM. Frequency-domain HRV parameters were evaluated using DC-01-ECG software (version 2.1.0.7, SEM), in accordance to the Task Force of the European Society of Cardiology and the North American Society of Pacing and Electrophysiology (1996).

The values of low-frequency (LF; 0.04–0.15 Hz) and high frequency (HF; 0.15–0.40 Hz) band, as well as LF/HF ratio, were calculated for 5-min intervals. Three daytime fasting intervals (08:00–08:05, 13:00–13:05 and 17:00–17:05), three postprandial intervals (10:30–10:35, 15:30–15:35 and 19:30–19:35) and one nocturnal interval (03:00–03:05) were analyzed. In subjects with CGM-defined hypoglycemic events, HRV parameters in 5-min recordings, matching the lowest levels of interstitial glucose, were also estimated. In these patients fasting, postprandial and nocturnal HRV values were assessed for hypoglycemia-free time.

Cardiovascular autonomic neuropathy (CAN) was revealed by standardized autonomic function tests, including response to standing, deep breathing, and Valsalva maneuver (Spallone et al. 2011a).

### Ethical issues

The study was conducted in accordance with the principles of the Declaration of Helsinki and the International Conference on Harmonization Good Clinical Practice guidelines. The protocol of the study was approved by institutional ethic committee. Written informed consents were obtained from all patients prior to the study procedures.

### Statistical analysis

Data are presented as medians, 25th and 75th percentiles, unless stated otherwise. STATISTICA 64 10 (StatSoft.Inc, USA) was used for statistical processing of data (StatSoft. Inc. 2011). The significance of differences between groups was assessed by ANOVA Kruskal–Wallis, Mann–Whitney test or Wilcoxon matched-pair test when appropriate. Spearmen rank correlation analysis was applied for assessment of the relationships between studied parameters. The multiplestepwise regression analysis was used to estimate the significance of clinical and GV parameters as predictors of HRV.

## Results

Clinical characteristics of participants are summarized in Table 1. The profile of cardiovascular disease and risk factors included arterial hypertension (n = 66), dyslipidemia (n = 63), obesity (n = 62), CAN (n = 46), chronic kidney disease (n = 42), and coronary artery disease (n = 35, five patients with a prior myocardial infarction). Five women were current smokers. Patients were treated with angiotensin converting enzyme inhibitors (n = 36) or angiotensin receptor blockers (n = 29), in combination with diuretics (n = 39) and/or calcium channel blockers (n = 20). Thirty-five patients received beta-blockers. Other regular therapy included aspirin (n = 47) and statins (n = 49).

**Table 1:** Characteristics of patients

Parameter	Median	Q25; Q75
Age (years)	65	61; 67
Body mass index (kg/m^2^)	35	29.4; 38.8
Waist circumference (cm)	110	100; 120
Hip circumference (cm)	112.5	103; 121
Diabetes duration (years)	16	11; 20
Duration of insulin therapy (years)	6	3; 9
Daily insulin dose (U)	52	34; 68
Daily insulin dose (U/kg)	0.6	0.4; 0.7
HbA1c (%) (mmol/mol)	8.7 (72)	7.6; 9.4 (60; 79)
Total cholesterol (mmol/L)	5.0	4.5; 5.9
LDL cholesterol (mmol/L)	3.3	2.5; 4.0
HDL cholesterol (mmol/L)	1.4	1.2; 1.7
Triglycerides (mmol/L)	1.4	1.1; 1.8
Serum creatinine (μmol/L)	86.2	76; 99.8
eGFR (CKD-EPI 2009) (mL/min/1.73 m^2^)	62	53; 76

The data are shown as medians (25th; 75th percentiles)

*LDL* low-density lipoproteins, *HDL* high-density lipoproteins, *eGRF* estimated glomerular filtration rate

**Fig. 1:**
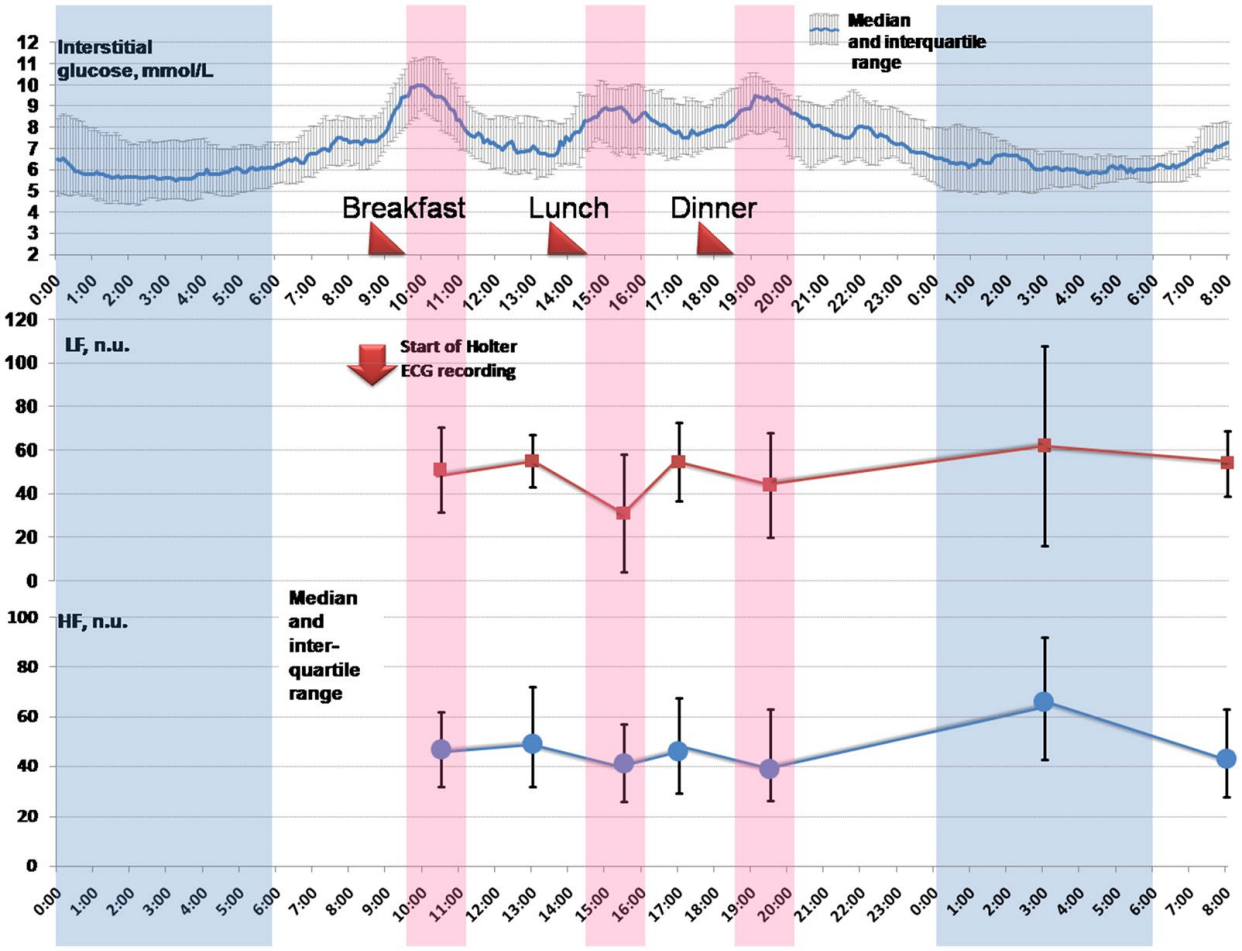
Daily fluctuations of interstitial glucose (*upper curve*), LF power (*middle curve*) and HF power (*lower curve*) in observed diabetic patients

The noticeable individual differences in HRV response to the meals were observed in diabetic subjects. Nonetheless, the mean power of LF component was diminished at postprandial state as compared to fasting one (Table 2). The HF component demonstrated a tendency to decrease after the meals, meanwhile LF/HF ratio showed no changes. Concordantly, daytime profile of HF and LF was reciprocal to that of interstitial glucose (Fig. 1). Both LF and HF values increased significantly at night compared to daytime fasting and postprandial periods.

**Table 2:** Diurnal profile of frequency-domain HRV parameters in observed diabetic patients

Parameter	Period		
	Day-time/fasting (1)	Day-time/postprandial (2)	Night (3)
LF (n.u.)	57 (40; 69)	46.7 (34.5; 60.0) *p*_1–2_ = *0.01*	62 (38; 108) *p*_1–3_ = *0.006* *p*_2–3_ = *0.0001*
HF (n.u.)	47.7 (30.0; 71.3)	42.7 (30.0; 62.3) *p*_1–2_ = *0.09*	66 (43; 92) *p*_1–3_ = *0.0001* *p*_2–3_ = *0.00002*
LF/HF	1.2 (0.9; 1.4)	1.1 (0.9; 1.3) *p*_1–2_ = *0.6*	1.0 (0.7; 1.4) *p*_1–3_ = *0.2* *p*_*2*–*3*_ = *0.6*

The data are shown as medians (25th; 75th percentiles). The differences were assessed by Wilcoxon matched pair test

*p*_*1–2*_ p-value for the differences between day-time/fasting period and day-time/postprandial one, *p*_*1–3*_ p-value for the differences between day-time/fasting period and night, *p*_*2–3*_ p-value for the differences between day-time/postprandial period and night

*HF* high-frequency domain, *HRV* heart rate variability, *LF* low-frequency domain, *n.u.* normalized units

As expected, the values of frequency-domain HRV measures were lower in patients with CAN as compared to those without (Table 3). The differences between two groups were significant for daytime fasting, postprandial and nocturnal LF values, as well as for nocturnal HF power. In patients with normal standard autonomic function tests, the values of LF and HF power, but not LF/HF ratio, declined markedly after the meals. In subjects with CAN the postprandial reduction in LF and HF power was diminished. Diurnal daytime/nocturnal HRV fluctuations were also blunted in subjects with CAN.

**Table 3:** Diurnal profile of frequency-domain HRV parameters in diabetic patients with and without CAN

Period	HRV parameter	Patients without CAN (n = 21)	Patients with CAN (n = 46)	p
Day/fasting (1)	LF (n.u.)	65.8 (55.7; 81.2)	48.3 (30; 65.3)	0.005
	HF (n.u.)	58.3 (35; 81)	43.7 (27.7; 63)	0.14
	LF/HF	1.2 (1.0; 1.4)	1.1 (0.9; 1.3)	0.06
Day/postprandial (2)	LF (n.u.)	51.6 (37; 76.3) *p*_1–2_ = *0.03*	43.3 (31.5; 54) *p*_1–2_ = *0.11*	0.05
	HF (n.u.)	49.8 (31.5; 67.0) *p*_1–2_ = *0.05*	39.7 (30; 58) *p*_1–2_ = *0.7*	0.14
	LF/HF	1.1 (0.9; 1.5) *p*_1–2_ = *0.17*	1.1 (0.9; 1.3) *p*_1–2_ = *0.58*	0.7
Night (3)	LF (n.u.)	88 (49; 114) *p*_1–3_ = *0.16* *p*_2–3_ = *0.03*	56 (37; 101) *p*_1–3_ = *0.03* *p*_2–3_ = *0.0007*	0.01
	HF (n.u.)	79 (50; 123) *p*_1–3_ = *0.01* *p*_2–3_ = *0.005*	64 (40; 75) *p*_1–3_ = *0.003* *p*_2–3_ = *0.001*	0.04
	LF/HF	1.1 (0.7; 1.4) *p*_1–3_ = *0.2* *p*_2–3_ = *0.17*	1.1 (0.7; 1.4) *p*_1–3_ = *0.75* *p*_2–3_ = *0.86*	0.69

The data are shown as medians (25th; 75th percentiles). The differences between groups were assessed by Mann–Whitney test. The differences between variables within one group were assessed by Wilcoxon matched pair test

*p*_*1–2*_ p-value for the differences between day-time/fasting period and day-time/postprandial one, *p*_*1–3*_ p-value for the differences between day-time/fasting period and night, *p*_*2–3*_ p-value for the differences between day-time/postprandial period and night

*CAN* cardiovascular autonomic neuropathy, *HF* high-frequency domain, *HRV* heart rate variability, *LF* low-frequency domain, *n.u.* normalized units

The values of LF, measured at daytime fasting state, inversely correlated with HbA1c levels (r = −0.32, p = 0.02), duration of insulin treatment (r = −0.33, p = 0.01), and daily insulin dose (r = −0.4, p = 0.002). Fasting HF did not correlate with these parameters. However, postprandial HF values demonstrated weak inverse correlations with duration of insulin treatment (r = −0.29, p = 0.03) and daily insulin dose (r = −0.4, p = 0.002).

Nocturnal LF and HF correlated with HbA1c negatively (r = −0.29, p = 0.04 and p = −0.3, p = 0.04, respectively). Nocturnal LF tended to be lower in patients receiving beta-blockers as compared to other participants, but the difference was not statistically significant: 60.5 (38; 94) versus 79 (58; 118) n.u., respectively, p = 0.06. Other HRV parameters seem to be unaffected by these agents. There were no significant differences in HRV parameters between women over 65 years and younger ones. Nocturnal HF only demonstrated negative correlation with age (r = −0.34, p = 0.02). The current smokers did not differ significantly from other participants by HRV parameters.

Twelve valid episodes of daytime hypoglycemia were registered during simultaneous CGM and Holter monitoring in 11 participants. The mean LF values during CGM-defined hypoglycemia tended to be lower as compared to fasting ones: 42.8 (34.5; 48.3) versus 58.5 (43; 67.5) n.u., respectively, p = 0.09. The values of HF during daytime hypoglycemia were similar to those at fasting: 49.3 (42; 50) versus 49.2 (28.3; 68), p = 0.88. Patients with CAN as compared to those without had diminished LF/HF ratio during daytime hypoglycemia: 0.8 (0.6; 1.4) versus 1.2 (1.0; 1.7) respectively, p = 0.04. Seven hypoglycemic episodes in six patients were recorded at night. The nocturnal HF, LF and LF/HF values were similar when registered at low and normal/elevated glucose level (all p > 0.4).

Expectedly, mean interstitial glucose and the values of all GV indices were significantly higher in the daytime as compared to nocturnal hours (Table 4). The values of most GV measures did not differ significantly in patients with and without CAN (Table 5).

**Table 4:** Day-time and nocturnal GV parameters in observed diabetic patients

Parameter	1st night^#^ (1)	Day* (2)	2nd night* (3)	p_1–2_	p_2–3_	p_1–3_
Mean glucose (mmol/L)	5.9 (5.1; 6.2)	8.3 (7.2; 8.9)	6.5 (5.3; 7.0)	<0.0001	<0.0001	0.07
SD (mmol/L)	0.5 (0.4; 1.0)	1.5 (1.2; 1.9)	0.7 (0.5; 0.9)	<0.0001	<0.0001	0.87
CONGA2 (mmol/L)	5.3 (4.3; 6.6)	7.1 (6.2; 7.7)	5.7 (4.9; 6.4)	<0.0001	<0.0001	0.10
LI (mmol/L)^2^/h	0.4 (0.1; 0.7)	2.1 (1.5; 3.3)	0.4 (0.2; 0.9)	<0.0001	<0.0001	0.51
J-index (mmol/L)^2^	14.4 (9.4; 22.9)	30.1 (24.8; 37.5)	15.9 (10.7; 20.5)	<0.0001	<0.0001	0.07
LBGI	0.7 (0.02; 2.9)	0.4 (0; 1.4)	0.6 (0.01; 1.7)	0.003	0.02	0.39
HBGI	0.5 (0; 1.8)	3.9 (2.9; 6.3)	0.4 (0.01; 1.6)	<0.0001	<0.0001	0.72
MAGE (mmol/L)	–	3.6 (2.7; 4.4)	–	–	–	–
MAG (mmol/L/h)	0.9 (0.7; 1.4)	1.8 (1.5; 2.4)	0.9 (0.5; 1.3)	<0.0001	<0.0001	0.07

The differences were assessed by matched pair Wilcoxon test

*CONGA2* 2-h continuous overlapping net glycemic action, *GV* glucose variability, *HBGI* high blood glucose index, *LBGI* low blood glucose index, *LI* lability index, *MAG* mean absolute glucose, *MAGE* mean amplitude of glucose excursions, *SD* standard deviation

* During Holter ECG

^#^Before Holter ECG

**Table 5:** Day-time and nocturnal GV parameters in diabetic patients with and without CAN

Parameter	Patients without CAN (n = 21)			Patients with CAN (n = 46)			p
	Median	Q25	Q75	Median	Q25	Q75	
1st night^#^ (1)							
Mean (mmol/L)	6.2	5.6	7.6	5.6	5.0	7.5	0.75
SD (mmol/L)	0.9	0.4	1.3	0.6	0.4	0.8	0.48
CONGA (mmol/L)	5.4	5.0	6.5	5.3	4.4	6.6	0.98
LI (mmol/L)^2^/h	0.5	0.2	0.7	0.4	0.1	0.7	0.91
J-index (mmol/L)^2^	17.8	11.6	22.1	12.9	10.6	20.7	0.81
LBGI	1.1	0.0	2.1	0.7	0.0	2.8	0.91
HBGI	1.5	0.0	1.8	0.4	0.0	1.6	0.53
M-value	1.4	0.6	2.8	3.3	0.6	6.1	0.75
MAG (mmol/L/h)	0.9	0.7	1.1	0.9	0.7	1.4	0.62
Day* (2)							
Mean (mmol/L)	7.9	7.0	8.7	8.3	7.5	8.8	0.48
SD (mmol/L)	1.7	1.4	2.1	1.5	1.2	1.9	0.09
CONGA (mmol/L)	6.8	6.2	7.3	7.1	6.6	7.7	0.43
LI (mmol/L)^2^/h	2.5	1.8	4.1	2.1	1.4	3.2	0.29
J-index (mmol/L)^2^	30.3	25.5	35.3	30.1	23.9	37.5	0.65
LBGI	0.4	0.4	4.7	0.4	0.0	0.9	0.13
HBGI	4.9	3.3	6.1	3.7	2.3	6.3	0.65
MAGE (mmol/L)	4.3	3.7	5.2	4.3	3.3	4.8	0.42
M-value	3.5	1.7	7.1	2.1	1.4	4.3	0.22
MAG (mmol/L/h)	1.6	1.5	2.2	1.9	1.5	2.2	0.80
2nd night* (3)							
Mean (mmol/L)	6.6	6.3	6.9	6.2	5.3	7.1	0.38
SD (mmol/L)	0.7	0.6	1.1	0.8	0.5	1.0	0.54
CONGA (mmol/L)	5.7	5.6	6.0	5.5	4.9	6.4	0.44
LI (mmol/L)^2^/h	0.5	0.4	0.6	0.4	0.2	0.8	0.64
J-index (mmol/L)^2^	17.8	15.2	20.2	15.5	10.8	21.8	0.45
LBGI	0.2	0.1	0.3	0.9	0.2	1.6	0.03
HBGI	0.7	0.3	1.8	0.3	0.0	1.8	0.40
M-value	0.3	0.1	0.4	1.1	0.5	2.1	0.01
MAG (mmol/L/h)	0.8	0.8	1.2	0.8	0.5	1.2	1.00

Between group differences assessed by Mann–Whitney test. The differences between variables were assessed by Wilcoxon matched-pair test

*CAN* cardiovascular autonomic neuropathy, *CONGA2* 2-h continuous overlapping net glycemic action, *GV* glucose variability, *HBGI* high blood glucose index, *LBGI* low blood glucose index, *LI* lability index, *MAG* mean absolute glucose, *SD* standard deviation

* During Holter ECG

^#^Before Holter ECG

Some daytime HRV parameters correlated with GV indices derived from CGM tracings, recorded during antecedent night. Specifically, daytime LF at fasting was inversely correlated with nocturnal MAG and LI (r = −0.48, p = 0.007, and r = −0.37, p = 0.05, respectively). Positive correlation was found between fasting LF and nocturnal LBGI (r = 0.39, p = 0.03). In the models of multiple regression analysis nocturnal MAG, but not other GV indices or clinical parameters (age, HbA1c, duration of insulin treatment, daily insulin dose), was associated with LF at daytime fasting (b = −0.54, R^2^ = 0.62, p = 0.01). The ratio of LF/HF at daytime fasting demonstrated positive correlation with nocturnal LBGI (r = 0.39, p = 0.03). The HF power at fasting correlated negatively with nocturnal MAG (r = −0.45, p = 0.01).

The LF power during daytime hypoglycemia demonstrated inverse correlations with nocturnal CONGA2 (r = −0.63, p = 0.04), J-index (r = −0.55, p = 0.05), HBGI (r = −0.58, p = 0.04) and MAG (r = −0.64, p = 0.04). The nocturnal HBGI and CONGA2, along with HbA1c and daily insulin dose, were predictors of LF during daytime hypoglycemia in multiple regression analysis (b = −1.4, b = −0.61, b = −1.24 and b = −0.73, respectively, R^2^ = 0.51, p = 0.008).

## Discussion

The obtained results demonstrate the intrinsic relationships between frequency-domain HRV parameters and interstitial glucose fluctuations in insulin-treated type 2 diabetic women. When glucose level fluctuates in the normal and hyperglycemic range, the mean power of LF and HF band, that reflects the sympathetic and vagal modulations predominately, tended to be reduced in postprandial state as compared to fasting state. Thereafter, daytime curves of HF and LF are reciprocal to that of interstitial glucose (Fig. 1). These data are concordant with those in non-diabetic subjects (Lu et al. 1999; Chang et al. 2010; Yoshizaki et al. 2014). Previously it was revealed that in healthy volunteers HF power is reduced within 60 min after the meal as compared to fasting state (Lu et al. 1999). In another study (Chang et al. 2010), the decrease in HF power from 40 to 120 min after the meals was demonstrated in healthy individuals; the power of LF component diminished from 60 to 120 min. Associations between diurnal 24-h rhythm in HRV and the timing and amount of meals were found recently in rotating shift workers (Yoshizaki et al. 2014). The question remains whether the food itself, glucose rise or both factors result in postprandial changes in sympathovagal balance in diabetic subjects.

We found that post-meal HRV fluctuations were blunted in patients with CAN. Nevertheless, nocturnal increase in HF and LF power was persisted in these patients. Unlike recently published data (Jun et al. 2015), we have not found any differences in the set of CGM-defined GV indices between diabetic patients with and without CAN.

Some data indicate that CAN and/or reduced HRV might be associated with the prevalence and severity of hypoglycemic events in diabetic subjects. In type 2 diabetic patients with coronary artery disease, hypoglycemic episodes were associated with depressed HRV (Infusino et al. 2010). Cardiovascular autonomic dysfunction predicted severe hypoglycemia in patients with type 2 diabetes in a 10-year follow-up study (Yun et al. 2014). In our sample, patients with CAN demonstrated diminished LF/HF ratio during daytime episodes of hypoglycemia. Impaired balance between the sympathetic and parasympathetic modulations during glucose nadirs may explain the association between CAN and hypoglycemia.

The concept of GV became a useful tool for unified description of the peaks and valleys in glucose concentration. The associations between HRV parameters and GV indices in type 2 diabetic patients were demonstrated in the previous studies. Thus, in women with newly diagnosed and well-controlled type 2 diabetes increased MAGE was associated with reduced cardiac autonomic modulation (Fleischer et al. 2015). Total power of HRV and most of time-domain HRV parameters were correlated negatively with SD and M-value in patients with type 2 diabetes treated with oral hypoglycemic agents (Kalopita et al. 2014). A significant association between nocturnal LF/HF ratio and 24-h MAGE was found in type 2 diabetic patients treated with diet and/or metformin (Di Flaviani et al. 2011). In this work we demonstrated for the first time that antecedent nocturnal glucose fluctuations, assessed by GV indices, are related to subsequent daytime HRV parameters at fasting state and during hypoglycemia. As already mentioned, both hyperglycemic and hypoglycemic fluctuations may affect HRV parameters in diabetic subjects. According to our results, nocturnal hypoglycemic stress, estimated by LBGI, is associated with a shift of the autonomic balance towards sympathetic activity (LF/HF ratio) on the next day. At the same time, nocturnal GV indices that reflect hyperglycemic fluctuations predominantly (CONGA2, J-index and HBGI) were associated negatively with sympathetic activity (LF component of HRV) during the episodes of subsequent daytime hypoglycemia. Nocturnal HBGI and CONGA2, along with HbA1c and daily insulin dose, were independent predictors for sympathetic activity at subsequent hypoglycemia. The results support the notion that both acute and chronic hyperglycemia can blunt autonomic response to hypoglycemia in type 2 diabetic subjects.

The limitation of our study is cross-sectional design that does not revealed the causality. Another obvious limitation is rather small sample size that did not reach desired one, estimated according to (Dupont and Plummer 1990). The study was performed in the hospital settings, i.e. in conditions that differ from free-living routine, but contribute to standardization of diet and daily activity. At the same time, we revealed for the first time the association of HRV with wide set of GV indices, reflecting the actual and antecedent glucose fluctuations.

Our findings underscore the impact of current and antecedent glucose fluctuations on HRV parameters. According to current recommendations, autonomic cardiovascular testing in diabetic subjects should be avoided during hypoglycemia and marked hyperglycemia (Spallone et al. 2011a). Apparently, in situation of unstable glycemic control, characterized by enhanced GV and/or abrupt glucose excursions, the results of short-term assessment of HRV should be interpreted with caution. Taking into account the effect of post-meal glucose rises on HRV, we recommend fasting state rather than postprandial one for HRV assessment.

## Conclusions

This study demonstrates the associations of frequency-domain HRV parameters with current and antecedent interstitial glucose fluctuations in insulin-treated type 2 diabetic women at high cardiovascular risk. In these patients, the postprandial glucose excursions are associated with reduction in sympathetic and parasympathetic activity. Nocturnal parameters of GV are related to next day sympathovagal modulations at fasting state and during hypoglycemia. Current and antecedent glucose fluctuations should be taken into consideration for the correct interpretation of HRV.

## Abbreviations

BMI: body mass index
CAN: cardiovascular autonomic neuropathy
CGM: continuous glucose monitoring
CKD: chronic kidney disease
CONGA2: 2-h continuous overlapping net glycemic action
eGFR: estimated glomerular filtration rate
GV: glycemic variability
HbA1c: glycated hemoglobin A1c
HBGI: high blood glucose index
HDL: high density lipoproteins
HF: high-frequency domain
HRV: heart rate variability
LBGI: low blood glucose index
LDL: low density lipoproteins
LF: low-frequency domain
LI: lability index
MAG: mean absolute glucose
MAGE: mean amplitude of glucose excursions
n.u.: normalized units
SD: standard deviation
